# Importance of basophil activation testing in insect venom allergy

**DOI:** 10.1186/1710-1492-5-11

**Published:** 2009-12-01

**Authors:** Mitja Kosnik, Peter Korosec

**Affiliations:** 1 University Clinic of Respiratory and Allergic Diseases, Golnik, Slovenia

## Abstract

**Background:**

Venom immunotherapy (VIT) is the only effective treatment for prevention of serious allergic reactions to bee and wasp stings in sensitized individuals. However, there are still many questions and controversies regarding immunotherapy, like selection of the appropriate allergen, safety and long term efficacy.

**Methods:**

Literature review was performed to address the role of basophil activation test (BAT) in diagnosis of venom allergy.

**Results:**

In patients with positive skin tests or specific IgE to both honeybee and wasp venom, IgE inhibition test can identify sensitizing allergen only in around 15% and basophil activation test increases the identification rate to around one third of double positive patients. BAT is also diagnostic in majority of patients with systemic reactions after insect stings and no detectable IgE. High basophil sensitivity to allergen is associated with a risk of side effects during VIT. Persistence of high basophil sensitivity also predicts a treatment failure of VIT.

**Conclusion:**

BAT is a useful tool for better selection of allergen for immunotherapy, for identification of patients prone to side effects and patients who might be treatment failures. However, long term studies are needed to evaluate the accuracy of the test.

## Introduction

Up to 0.1% of the population suffers from severe anaphylaxis after Hymenoptera insect sting. The prevalence is even higher in beekeepers, where can exceed 4% [1]. Venom immunotherapy (VIT) is the only effective treatment for prevention of serious allergic reactions to bee and wasp stings in sensitized individuals. However, there are still many questions and controversies regarding immunotherapy, like selection of the appropriate venom in patients with double positive tests or of patients with allergic reactions following European hornet stings, patients with negative sIgE and skin tests, detecting the patients at risk for side effects during immunotherapy and detecting the patients at risk of relapse after stopping immunotherapy [2].

## Patients with positive allergy tests to both honeybee and wasp venom

Up to 50% of patients with sting reactions have positive routine diagnostic tests (skin tests, specific IgE) to both honeybee and wasp venom. True double sensitization and cross-reactivity must be considered as a cause of the double positivity and diagnosed in this group of patients [3]. Cross-reactivity is possible on the protein level most often through venom hyaluronidases or through carbohydrates epitopes (CCD) [4,5]. Distinguishing between double sensitization and cross-reactivity is crucial for the choice of a proper allergen for specific immunotherapy in patients who didn't recognize the culprit insect [6]. Namely, patients should be treated with the venom, which induced sensitization. Immunotherapy with the venom to which a patient is not primarily sensitised can lead to an incomplete protection and treatment failure. On the other hand, treatment with a cross-reactive venom only or a mixture of venoms can lead to the formation of sIgE against epitopes to which the patient was not sensitised prior to immunotherapy [7,8].

If double sensitization is proven in a patient, who did not recognize the culprit insect, immunotherapy should be performed with both venoms; if cross - reactivity is the case, immunotherapy should be performed only with the venom that caused sensitization. Using specific IgE inhibition tests, Straumann was able to identify the insect that caused sensitisation in 4 out of 24 double positive patients [3]. We performed basophil activation tests (BAT) in 25 bee and wasp double positive patients and were able to characterize primary sensitization in one third of them (nearly all were found to be wasp allergic) [9]. BAT is a flow cytometry based test, which measures basophil activation markers like CD63 on surface of basophils after cells are stimulated in-vitro with allergen. We found some additional benefit of BAT over sIgE as basophils are not activated by clinically unimportant sIgE antibodies against CCD. BAT was shown to have higher specificity compared to sIgE, retaining higher sensitivity compared to skin tests. Moreover, the BAT test was feasible also in patients with very low level of sIgE, where inhibition tests were not possible.

## Immunotherapy of patients with allergic reactions following European hornet stings

In Europe, wasp stings are responsible for most Vespidae venom allergic reactions and only occasional reactions are caused by European hornet *(Vespa crabro) *stings. However, those reactions are very likely to be severe: the relative risk for life-threatening reactions after a *Vespa crabro *sting is about three times higher than it is for a honeybee or yellow jacket sting [10]. Those patients usually have positive skin tests and specific IgE to all Vespoidea venoms (*Vespula germanica, Vespa crabro *and also paper wasp *[Polistes])*.

In order to distinguish primary sensitisation from cross-reactivity, we performed cross inhibition tests in 24 consecutive patients who experienced anaphylactic reaction after European hornet stings: 17/24 patients were sensitised with only wasp *(Vespula germanica) *venom, 2/24 with completely cross-reactive epitopes, 1 with only European hornet venom and 4 with separate epitopes of both venoms [11]. We concluded that in Europe at least 70% of patients that experienced a systemic allergic reaction after European hornet stings were actually allergic to wasp venom. The logical conclusion from this observation would be that *Vespula germanica *venom remains the most appropriate immunotherapeutic agent for the majority of those patients.

## Anaphylaxis in patients with negative allergy tests

Although sIgE are believed to be the cause of allergic reactions after Hymenoptera insect stings, around 4% of patients with repeated systemic reactions and no detectable IgE [12,13]. Current guidelines for VIT suggest that immunotherapy should be performed only in patients with an IgE-mediated systemic reaction [6], but opinions about the diagnosis and treatment of patients with systemic reaction without IgE vary widely [2]. Some have proposed submitting every patient with a history of Hymenoptera sting allergy and negative allergy tests to a provocation test [14]. Negative skin test and no specific IgE may indicate a non-allergic reaction or a limited diagnostic sensitivity of the test. An alternative mechanism that could activate mast cells in the absence of sIgE is complement activation and the generation of anaphylatoxin C5a [15,16].

However, we found that 75% of 47 sIgE negative patients had a positive reaction in the flow cytometry based basophil activation test [17]. Even better results were shown by Ebo, who was able to identify sensitisation with a BAT test in 7 out of 7 sIgE negative patients [18]. The limitation of those studies is that due to ethical reasons the clinical history and not a sting challenge was used as a gold standard. However it has been shown that BAT test very rarely gives false positive results [19].

## Safety of VIT

In different immunotherapy protocols a cumulative dose of 100 μg of venom (corresponding to 2 honeybee or 10 wasp stings) is reached in few hours or days with no allergic reactions in the majority of patients, however at least 15% of patients exhibit systemic allergic reactions [20,21]. We investigated whether adult patients prone to systemic reaction during immunotherapy could be identified on the basis of basophil sensitivity to allergen [22]. We expressed the sensitivity as a ratio between basophil response to two concentrations of allergen. The first concentration (0.1 μg/ml) was shown in previous experiments as submaximal, eliciting only a partial activation of basophils in majority of tested subjects, and the second concentration (1 μg/ml) was maximal and elicited a complete activation of basophils in all responding subjects (it has to be stressed, that basophils of about 5% of patients do not respond at all to stimulation, and those patients are not suitable for BAT test). For each patient we calculated the ratio between basophil CD63 expression after stimulation with allergen in a concentration 0.1 and 1 μg/ml (0.1/1 ratio). Twelve out of 34 patients had reaction to VIT. In those 12 patients median 0.1/1 ratio was 0,99 (range: 0.17-1.95) Side-effects occurred in all patients with 0.1/1 ratios over 0.92. In contrast, in 22 patients with no side effects, the median 0.1/1 ratio was 0.25 (range: 0.02-0.92). These concentration-dependent activation ratios were significantly different between the groups with and without side reactions (P < 0.0001). Our results suggest that high basophil sensitivity to allergen is significantly associated with a risk of side effects to VIT. Similar results were obtained also in the children [23]. In the same study we showed that an elevated basal tryptase level was not a predicting factor for side effects of VIT [22].

## Effectiveness of VIT

Rush immunotherapy is very effective. Nearly complete tolerance after only a few days of VIT has been confirmed [24]. Immunotherapy is associated with an improved quality of life [25,26]

Long-term effectiveness after stopping the treatment is less reliable: in a Swiss study, 16% of bee allergic patients and 7.5% of wasp allergic patients treated for 3 to 7 years developed systemic reactions after stopping VIT; most reactions were mild, but there was a tendency for an increase in the severity of reactions after repeated re-stinging [27]. Moreover, a fatal reaction 9 years after the discontinuation of immunotherapy was recently described [28]. Some risk factors for relapse after immunotherapy are recognized [20]:

• Bee venom allergy

• Severe pre-treatment reaction

• Reaction to VIT injection

• Reaction during VIT

• Duration of VIT < 5 years

• Repeated re-stings after stopping VIT

Patients with reactions during immunotherapy are encouraged to receive immunotherapy indefinitely.

In our survey 229 patients treated with VIT between 1984 and 2004 were sent a questionnaire inquiring whether they had been stung by an insect to which the VIT had been directed [29]. 79% received VIT for more than 3 years and 55% were stung after discontinuing VIT. At the time of the first sting after stopping VIT, 8 (8%) had a systemic reaction. There were 40 patients who were stung more than once after ending VIT, among whom 7 (17.5%) experienced reactions of greater severity with the subsequent stings. All patients reported that their reactions after ending VIT were milder than before treatment. The likelihood of systemic reactions to stings was almost identical in patients treated for either more than or less than 3 years with VIT. Furthermore, patients who reacted after discontinuation of immunotherapy had higher basophil sensitivity (the sensitivity was comparable to a group of patients without immunotherapy) compared to a group of protected patients [30].

## Conclusion

Venom immunotherapy is very, but not completely, effective. However, managing patients with venom hypersensitivity has become less straightforward than it seemed to be some time ago. Diagnostic tests may be misleading and could pose problems regarding the selection of the appropriate venom for immunotherapy. The mechanisms of tolerance during first days of VIT are still to be documented. The long-term effectiveness of VIT is questionable. The basophil activation test appears to be a useful tool for better selection of allergen for immunotherapy, for identification of patients prone to side effects and patients who might be treatment failures. However, more long term studies are needed to evaluate the accuracy of the test.

## Competing interests

The authors declare that they have no competing interests.

## Authors' contributions

MK has been involved in drafting the manuscript, PK revisited the manuscript critically for important intellectual content. Both authors read and approved the final manuscript.
